# Linkage disequilibrium in Brazilian Santa Inês breed, *Ovis aries*

**DOI:** 10.1038/s41598-018-27259-7

**Published:** 2018-06-11

**Authors:** Amanda Botelho Alvarenga, Gregori Alberto Rovadoscki, Juliana Petrini, Luiz Lehmann Coutinho, Gota Morota, Matthew L. Spangler, Luís Fernando Batista Pinto, Gleidson Giordano Pinto Carvalho, Gerson Barreto Mourão

**Affiliations:** 1 Department of Animal Science, University of São Paulo (USP)/Luiz de Queiroz College of Agriculture (ESALQ), Piracicaba, SP Brazil; 2 Department of Animal Science, University of Nebraska, Lincoln, NE USA; 3 Department of Animal Science, Federal University of Bahia (UFBA), Salvador, BA Brazil

## Abstract

For genomic selection to be successful, there must be sufficient linkage disequilibrium between the markers and the causal mutations. The objectives of this study were to evaluate the extent of LD in ovine using the Santa Inês breed and to infer the minimum number of markers required to reach reasonable prediction accuracy. In total, 38,168 SNPs and 395 samples were used. The mean LD between adjacent marker pairs measured by r^2^ and |D′| were 0.166 and 0.617, respectively. LD values between adjacent marker pairs ranged from 0.135 to 0.194 and from 0.568 to 0.650 for r^2^ for |D′| across all chromosomes. The average r^2^ between all pairwise SNPs on each chromosome was 0.018. SNPs separated by between 0.10 to 0.20 Mb had an estimated average r^2^ equal to 0.1033. The identified haplotype blocks consisted of 2 to 21 markers. Moreover, estimates of average coefficients of inbreeding and effective population size were 0.04 and 96, respectively. LD estimated in this study was lower than that reported in other species and was characterized by short haplotype blocks. Our results suggest that the use of a higher density SNP panel is recommended for the implementation of genomic selection in the Santa Inês breed.

## Introduction

Genomic information is currently used in animal breeding programs to enable selection for difficult to measure traits, increase the overall rate of genetic gain, and to improve the understanding of genetic and biological causes underlying phenotypic variation. Genomic selection (GS) is an approach which uses genome-wide markers simultaneously to predict breeding values^1^. This approach has been shown to increase the rate of genetic gain when pedigree-based selection is suboptimal^1^, which is the case for lowly heritable traits. For instance, GS based on simulated data showed an increase in reliability of breeding values for young animals when using genomic (r^2^ > 60%) versus parent average (r^2^ = 32%) information, equivalent to approximately 20 offspring^2^. Furthermore, genetic gain can be increased using genomic information by shortening the generation interval^1^. Alternatively, genetic markers scattered across the genome offer an opportunity to conduct genome-wide association studies (GWAS) to characterize genes underlying genetic variation for traits of interest.

The success of GS and GWAS are dependent on linkage disequilibrium (LD) or gametic disequilibrium between the markers and causal mutations^3^ because generally only the markers are observed and the casual mutations are unknown. The LD between a marker and a causal mutation can be considered as the proportion of causal mutation variance that can be captured by the marker variance^4,5^. Through the knowledge of the degree of LD, it is possible to define the density of genetic markers necessary to achieve a certain accuracy of prediction and to determine when the estimates of genetic marker effects should be updated. It has been well documented that simply increasing marker density does not improve prediction accuracies. Although increased marker density improves resolution, it can also decrease power and add noise to the analyses by the use of non-informative SNP. Furthermore, increased marker density can dilute individual marker effects if, for example, two markers are associated with the same QTL and the two markers are in high LD with each other.

LD is defined as a non-random association between alleles at different loci^6^, and it is commonly represented by |D′| and r^2^ metrics^7^. The extent of LD can vary between and within species due to evolutionary history and population structure mainly characterized by insertions, deletions, chromosomal rearrangements, or inversions^4^. This association between markers and causal mutations may change overtime due to recombination and selection^4^ necessitating the re-estimation of marker effects.

Estimates of LD have been reported in ovine for some domestic pure and crossbred populations, as well as in wild sheep by using microsatellites and SNP markers^4,8–14^. Nevertheless, there are few studies that report LD estimates for Brazilian Santa Inês sheep using SNP. Ovine populations have retained a relatively high level of genetic diversity, unlike bovine, which justify the importance of LD mapping in many breeds within species^15^. Moreover, LD estimates between different breeds can be informative relative to the overall diversity level in a species and the selection level applied to them.

Therefore, the aim of the current study was to characterize LD structure in Brazilian Santa Inês sheep for the first time, given its commercial importance for meat production, reproductive efficiency, and tropical adaptation in Brazil, and compare the LD observed in the Santa Ines breed with other breeds. Beynon *et al*.^16^ mentioned the importance of studies focused on breeds as a chance to identify variation and understand the biological mechanisms that enable these breeds to survive in different local environments.

Many studies have evaluated imputation accuracy^17^ and the accuracy of genomic estimated breeding values using different marker panel densities in sheep^18–20^. The appropriate panel density could be specific to each species and breed depending on overall LD structure. Unfortunately, the current genotyping costs in sheep are greater than the economic value of breeding animals^21^. Consequently, we also aimed to provide an estimate of the marker density required for genomic studies in the Santa Inês breed.

## Results and Discussion

### Descriptive statistics

After quality control (QC), 38,168 autosomal SNPs remained comprising approximately 53% of the entire panel. The SNPs retained after QC spanned a total of 299.63 megabases (Mb) of the genome, with a mean (standard deviation) distance between adjacent SNP of 0.07 (0.075) Mb. This value was close to that obtained by Liu *et al*. in Spanish Churra sheep (0.06 Mb)^14^. SNPs were evenly distributed throughout the genome as the distances between adjacent markers ranged from 0.064 to 0.085 Mb. The chromosomes differ in size and SNP quantity, with chromosome 24 being the smallest in size - OAR24 (44.21 Mb). Liu *et al*.^14^ observed a similar behavior considering the same SNP panel (OAR24- 44.85 Mb), with OAR24 being the smallest chromosome (44.85 Mb) whereas the OAR2 was the largest (263.11 Mb). The number of SNPs per chromosome was proportional to the size of each chromosome. Descriptive statistics of the SNP and LD (r^2^ and |D′|) for each chromosome are presented in Table 1.

**Table 1:** Descriptive analyses, MAF, F, N_e_,and average linkage disequilibrium (r^2^ and |D′|) between adjacent and all pairwise SNP pairs by chromosome.

Chr	Size (Mb)	N° ${{\rm{SNPs}}}_{f}$	Dist. (Mb)	MAF	F	${N}_{e}$	r^2^ pairwise SNP	r^2^ adjacent SNP	|D′| pairwise SNP	|D′| adjacent SNP
1	243.8	4392	0.0676	0.2917	0.036(0.0373)	4530	0.010(0.0238)	0.172(0.2190)	0.176(0.1775)	0.625(0.3353)
2	263.1	4020	0.0655	0.2916	0.157(0.0381)	3196	0.011(0.0256)	0.192(0.2416)	0.177(0.1808)	0.639(0.3310)
3	242.5	3606	0.0673	0.2895	0.045(0.0640)	1491	0.011(0.0264)	0.183(0.2306)	0.181(0.1857)	0.650(0.3368)
4	127.0	1976	0.0643	0.2907	0.067(0.0569)	1276	0.016(0.0339)	0.181(0.2324)	0.215(0.2065)	0.639(0.3373)
5	115.9	1723	0.0673	0.2865	0.060(0.0660)	1303	0.015(0.0334)	0.169(0.2236)	0.215(0.212)	0.638(0.3376)
6	129.0	1979	0.0652	0.2862	0.062(0.0642)	1068	0.014(0.0301)	0.155(0.2047)	0.213(0.2072)	0.611(0.3319)
7	108.5	1664	0.0653	0.2934	0.059(0.0544)	1526	0.015(0.0314)	0.167(0.2192)	0.203(0.1984)	0.612(0.3363)
8	97.7	1521	0.0643	0.2920	0.051(0.0473)	1616	0.016(0.0334)	0.165(0.2220)	0.214(0.2062)	0.595(0.3429)
9	100.7	1539	0.0655	0.2879	0.050(0.0519)	1841	0.018(0.0371)	0.166(0.2214)	0.222(0.2094)	0.619(0.3340)
10	94.0	1319	0.0714	0.2872	0.045(0.0415)	3881	0.020(0.0427)	0.191(0.2507)	0.237(0.2203)	0.638(0.3340)
11	66.8	860	0.0778	0.2864	0.043(0.0357)	3409	0.017(0.0358)	0.152(0.2109)	0.230(0.2229)	0.614(0.3382)
12	86.0	1245	0.0692	0.2907	0.042(0.0388)	3742	0.017(0.0361)	0.157(0.2096)	0.221(0.2118)	0.622(0.3341)
13	88.8	1214	0.0733	0.2917	0.041(0.0382)	3707	0.017(0.0351)	0.169(0.2285)	0.213(0.2027)	0.603(0.3407)
14	68.6	836	0.0823	0.2868	0.039(0.0354)	3173	0.017(0.0362)	0.157(0.2090)	0.227(0.2187)	0.609(0.3373)
15	89.8	1223	0.0735	0.2932	0.040(0.0358)	3605	0.017(0.0363)	0.169(0.2246)	0.225(0.2187)	0.636(0.3366)
16	77.0	1090	0.0708	0.2668	0.045(0.0404)	3793	0.022(0.049)	0.194(0.2423)	0.256(0.2329)	0.650(0.3183)
17	78.4	1070	0.0734	0.2918	0.044(0.0409)	3431	0.018(0.0376)	0.155(0.2147)	0.226(0.2133)	0.602(0.3405)
18	71.8	1011	0.0711	0.2835	0.043(0.0410)	3532	0.018(0.0371)	0.160(0.2143)	0.232(0.2201)	0.622(0.3401)
19	64.7	887	0.0731	0.2904	0.042(0.0381)	3302	0.019(0.0384)	0.172(0.2211)	0.236(0.2216)	0.623(0.3284)
20	55.3	818	0.0678	0.2910	0.063(0.0631)	1386	0.022(0.0419)	0.148(0.1893)	0.251(0.2270)	0.620(0.3295)
21	55.0	654	0.0843	0.3001	0.074(0.0768)	1464	0.023(0.0233)	0.157(0.2142)	0.244(0.2223)	0.583(0.3384)
22	54.9	758	0.0725	0.2902	0.049(0.0423)	1638	0.021(0.0210)	0.173(0.2226)	0.245(0.2300)	0.641(0.3311)
23	66.2	835	0.0794	0.2878	0.049(0.0423)	1113	0.020(0.0203)	0.142(0.1963)	0.236(0.2142)	0.585(0.3329)
24	44.2	524	0.0845	0.2925	0.035(0.0364)	1439	0.020(0.0209)	0.135(0.1972)	0.240(0.2243)	0.568(0.3391)
25	48.0	731	0.0658	0.2890	0.072(0.0690)	1689	0.022(0.0225)	0.166(0.2191)	0.248(0.2233)	0.602(0.3323)
26	49.7	673	0.0740	0.2938	NA	1149	0.022(0.0224)	0.165(0.2138)	0.244(0.2258)	0.611(0.3333)

Chr: chromosome; Size (Mb): size of chr in mega pair base; N° ${{\rm{SNPs}}}_{f}$: SNP count after quality control for each chr; Dist. (Mb): mean intermarker adjacent distance; MAF: mean of minor allele frequency on each chr; F: inbreeding coefficient; ${N}_{e}$: effective population size; r^2^ pairwise SNP: mean (standard deviation) r^2^ estimated for each pairwise combination of SNPs on each chromosome; r^2^ adjacent SNP: mean r^2^ between adjacent SNPs; |D′| pairwise: mean (standard deviation) |D′| estimated for each pairwise combination of SNPs on each chromosome; |D′| adjacent SNPs: mean |D′| between adjacent SNPs.

In addition, 35% of the SNPs (18,716) had minor allele frequency (MAF) lower than 0.20, with a mean MAF over all SNPs of 0.35. According to another sheep study, 33% of the SNPs had MAF lower than 0.20^22^. Extending our comparison to other species, the mean MAF was relatively higher than those found for Bos taurus indicus, with values ranging from 0.19 to 0.25^23,24^. The MAF is important because LD, independent of the metric used, is a function of allelic frequency. In general, low MAF may correspond to a larger difference in allele frequency of coupled alleles, which can result in lower estimates of LD as measured by either r^2^ or |D′|^25^. Consequently, applying QC and the choice of QC criteria can affect the distribution and extent of LD^6^.

### Inbreeding coefficient and effective population size

For a better understanding of the population described in this study, inbreeding coefficient (F) and effective population size (*N*_*e*_) were estimated for all chromosomes together and for each chromosome separately, using genomic information. The estimate of F was 0.04, a relatively low coefficient for a population that originated from the same commercial herd. Using pedigree information to estimate the inbreeding coefficient, Pedrosa *et al*. found values equal to 0.02 in the Santa Inês breed^26^. Al-Mamun *et al*. found average inbreeding coefficients for Merino, Border Leicester and Poll Dorset equal to −0.013, 0.09 and 0.02, respectively^13^. A recently published study in ovine found average inbreeding coefficients based on excess of homozygosity (standard deviation- SD) of −0.008 (0.031), ranging from −0.079 to 0.301^12^. Compared with Kijas *et al*.^11^ and Liu *et al*.^14^, the F estimated from the Santa Inês breed was lower. Negative inbreeding coefficients occur when the number of observed homozygous loci is lower than the expected, suggesting that the population is more heterogeneous than expected, perhaps due to the composite nature of the breed.

In the *N*_*e*_ estimation process, genetic distance between markers was estimated by a fixed ratio across the whole genome of one Mb per centiMorgan (cM). Prieur *et al*. evaluated three different methods to transform the genetic distance in ovine, and concluded that the estimation process using CRIMAP software (v2.503) was more accurate^27^. However, Prieur *et al*. also verified that the ranking for r^2^ and *N*_*e*_ between breeds were not affected by the method used and mentioned that the LD estimator was not different between methods^27^.

The *N*_*e*_ estimated herein was 96 in the current generation. Kijas *et al*.^15^ observed *N*_*e*_ equal to 520 in the Brazilian Santa Inês breed, however, in their study only 47 animals were used. Pedrosa *et al*. also estimated *N*_*e*_ using pedigree information and found a relatively low value (76) in Santa Inês^26^. These differences in *N*_*e*_ can be due to the number of animals used (395 vs. 47 vs. 17,097) and the source of relationship information (genomics vs. pedigree). Al-Mamun *et al*. found values of *N*_*e*_ ranging from 140 (Border Leicester breed) to 348 (Merino breed)^13^. Brito *et al*.^12^ found values of *N*_*e*_ in the most current generations in multi-breed sheep populations ranging from 125 to 974. Using a Spanish Churra sheep population, García-Gámez *et al*.^28^ and Chitneedi *et al*.^29^ estimated *N*_*e*_ equal to 159 and 83, respectively.

The presence of artificial selection in the population under study was verified through the reduction of *N*_*e*_ over the generations. In this study, *N*_*e*_ ranged from 1,705 to 28,191 between 16 and 296 generations, respectively, before the current generation. Mastrangelo *et al*. estimated the *N*_*e*_ at 295 generations ago to be 747 animals in Barbaresca sheep^30^. Liu *et al*. observed *N*_*e*_ equal to 4,472 and 160 at 2,000 and 5 generations ago, assuming that one Mb is equivalent to one cM^14^. Brito *et al*.^12^ reported estimates of effective population size of 5,537 animals 1,000 generations ago to 687 in the most recent generation. We hypothesize that the large difference in *N*_*e*_ between the current and historic generations could be because the breeds that comprise the composite breed of Santa Inês were divergent historically and, thus, these estimates include multiple divergent breeds. The Santa Inês breed is relatively new, having only begun in the 1950s by non-systematic crossing of the Brazilian Somali, Bergamasca and Morada Nova breeds^31^. This illustrates that the large estimates of historic *N*_*e*_ reflect time points before the formation of the breed, and even before the domestication of ovine.

We also estimated the *N*_*e*_ for each chromosome. Chromosome 6, OAR6, exhibited the smallest *N*_*e*_, which was in contrast to the results of Liu *et al*. that reported the smallest *N*_*e*_ for OAR10^14^.

### Linkage disequilibrium analysis between adjacent SNPs

The average (SD) r^2^ and |D′| values estimated between adjacent SNPs from the 26 autosomal chromosomes were 0.166 (0.2189) and 0.617 (0.3349), respectively. Using the dairy sheep breed Frizarta, Kominakis *et al*. estimated r^2^ and |Dʹ| equal to 0.18 and 0.50, respectively, at an average inter-marker distance of 0.031 Mb^32^. Mastrangelo *et al*. observed average r^2^ (SD) in Sicilian sheep equal to 0.155 (0.2040)^33^. Al-Mamun *et al*. also reported LD estimates from multiple domesticated sheep (*Ovis aries*) breeds including: Merino (MER), Border Leicester (BL), Poll Dorset (PD) and crossbred populations (i.e., F_1_ crosses of Merino and Border Leicester (MxB) and MxB crossed to Poll Dorset (MxBxP)). The authors used the same genotype panel but adopted a different data quality control (MAF < 0.01) and reported a mean r^2^ of 0.12 (MER), 0.20 (BL), 0.19 (PD), 0.13 (MxB) and 0.13 (MxBxP); and mean |D′| of 0.52 (MER), 0.72 (BL), 0.69 (PD), 0.54 (MxB) and 0.55 (MxBxP)^13^. In the Barbaresca sheep breed, the mean r^2^ across autosomes was 0.215, with an average distance between adjacent SNP pairs of 0.063 Mb^30^.

A study published with multi-breed sheep reported mean (SD) r^2^ of 0.26 (0.100)^12^. The estimates of r^2^ are relatively consistent across sheep populations, with the exception of larger r^2^ values reported by Brito *et al*. Nevertheless, we should consider that the distance between markers was much shorter in Brito *et al*. than herein (4.74 kb versus 70 kb in the present study), which can be one reason for the increase in r^2^. Additionally, Brito *et al*. reported LD levels less than 0.10 for SNP located more than 0.04 Mb apart^12^. A recent study from Michailidou *et al*. observed a mean r^2^ equal to 0.121, 0.098, and 0.092 in Boutsko, Chios, and Karagouniko, respectively, with the average intermarker distance 0.27 Mb for all breeds^34^.

Sheep populations have been associated with lower levels of LD in comparison to other ruminant and nonruminant species. Although the comparison between species is difficult due differences in genome size as well as the quality control applied, mean values between adjacent SNPs of 0.32 (r^2^) and 0.69 (|D′|) were estimated from the Australian Holstein-Friesian cattle population using 9,195 SNP with the mean SNP distance equal to 0.25 Mb^6^. The mean r^2^ for pigs of Landrace (87 animals), Yorkshire (96 animals), Hampshire (78 animals) and Duroc (90 animals) breeds were 0.36, 0.39, 0.44, and 0.46 estimated from 40, 144, 39, 110, 32, 370 and 34,129 SNP spaced at average distances of 0.06, 0.06, 0.07, and 0.07 Mb, respectively^35^.

The average LD (SD) between adjacent SNP within the same chromosome ranged from 0.135 (0.1972) to 0.194 (0.2423) for r^2^ and 0.568 (0.3391) to 0.650 (0.3368) for |D′| (Table 1). Chromosomes 6, 11, 12, 14, 17, 20, 21, 23 and 24 had lower average LD using r^2^ lower than the 0.16 threshold^24^. Considering r^2^ metrics between adjacent SNPs, chromosomes 2, 10 and 16 had higher levels of LD compared to other chromosomes. The high level of LD present on OAR10 was similar to that observed by Al-Mamun *et al*.^13^.

### Linkage disequilibrium analysis among all pairwise SNPs

The average (SD) for r^2^ and |D′| estimated between all pairwise SNPs on the 26 autosomal chromosomes were 0.018 (0.032) and 0.225 (0.213), respectively. In a study which used microsatellite markers to evaluate LD using chromosomes 1–10 of domestic sheep (*Ovis aries*) with mean distance between markers ranging from 10 to 40 Mb, a mean (SD) value of 0.211 (0.004) for |D′| was estimated^10^. Al-Mamun *et al*. who also used domesticated sheep (*Ovis* aries), found mean r² between all pairwise SNPs (0.05 Mb mean distance) of 0.007 (MER), 0.013 (BL), 0.018 (PD), 0.009 (BxM) and 0.012 (BxMxP); and mean |D′| of 0.168 (MER), 0.29 (BL), 0.27 (PD), 0.18 (BxM) and 0.19 (BxMxP)^13^. Additionally, Miller *et al*. using non-domesticated sheep (*Ovis canadensis* and *Ovis dalli*) and the same genotype panel but adopting a different QC (MAF < 0.10), reported a mean r^2^ (SD) of 0.042 (0.067)^4^. Considering the confidence interval obtained for the estimates presented in this study as well as in the studies previously reported, it is possible to assume that estimates of r^2^ and |D′| across all SNP combinations on a chromosome are relatively consistent across sheep populations.

**Figure 1:**
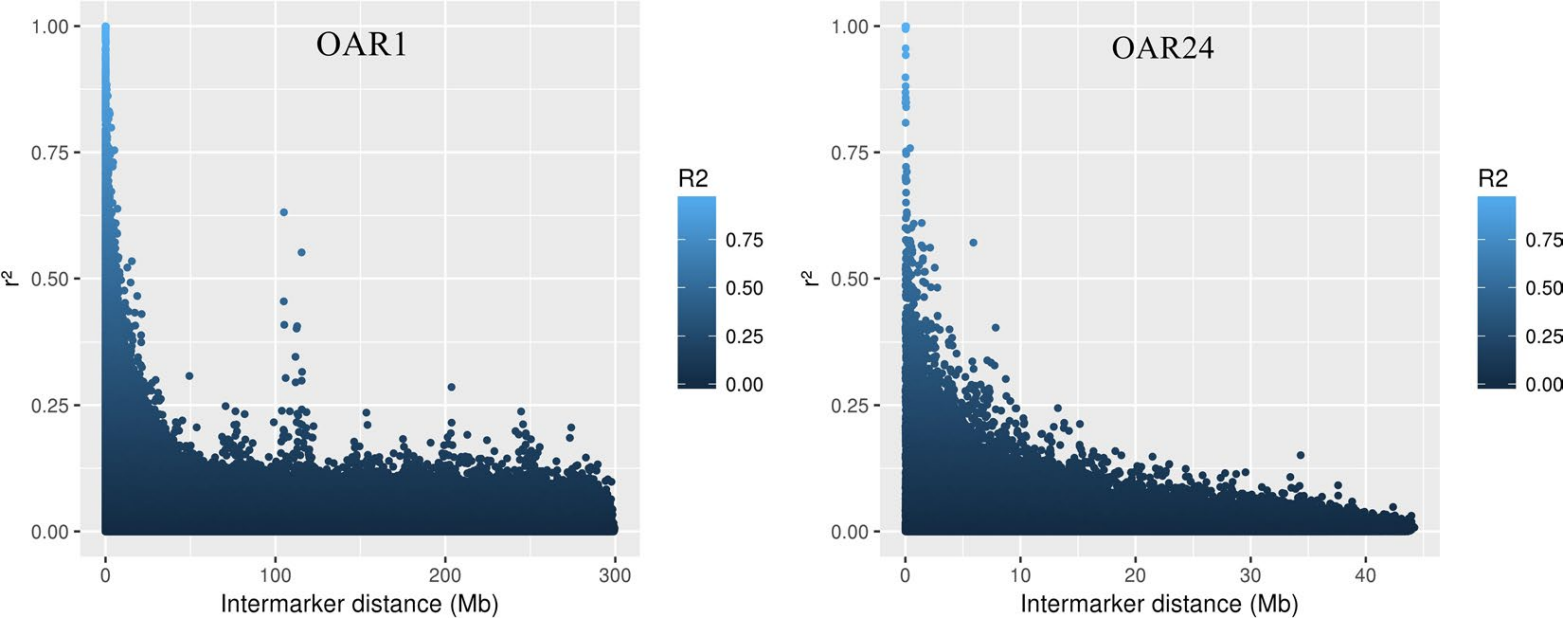
Linkage disequilibrium (LD) measured by r^2^ plotted as a function of intermarker distance (Mb) for chromosomes 1 (OAR1) and 24 (OAR24).

**Figure 2:**
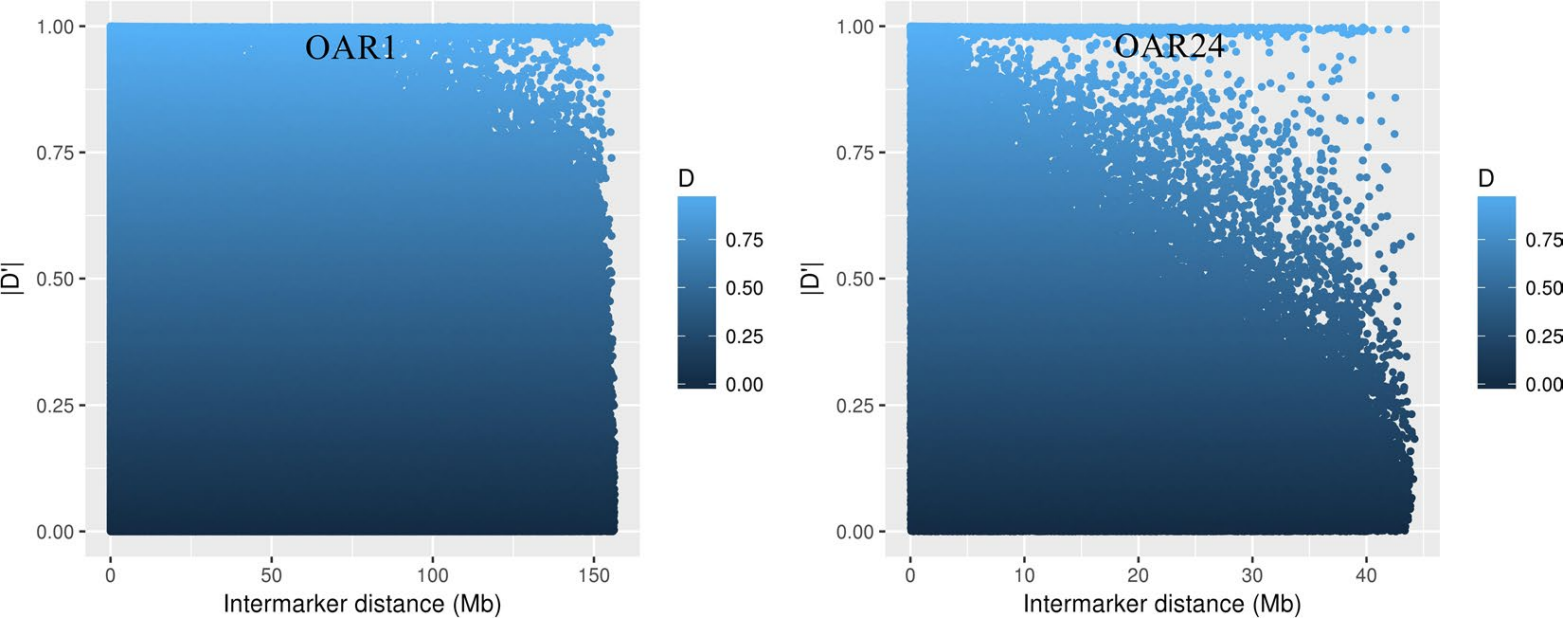
Linkage disequilibrium (LD) measured by |D′| plotted as a function of intermarker distance (Mb) for chromosomes 1 (OAR1) and 24 (OAR24).

Figures 1 and 2 illustrate r^2^ and |D′|, respectively, as a function of the intermarker distance for chromosomes 1 and 24. Supplementary Fig. S1 and S2 depict r^2^ and |D′|, respectively, for the other chromosomes. Overall, the relationship between LD and intermarker distance suggest that as intermarker distance decreases, LD increases. A notable exception is chromosome 1. On this chromosome, r^2^ presented secondary high peaks around the interval from 100 to 150 Mb (Fig. 1). On all chromosomes, |D′| maximum was observed between many SNP pairs with high intermarker distances (Fig. 2). We contend that this might occur due to the dependence of |D′| on allele frequency. The unexpected increase in LD between some SNP pairs with larger intermarker distances could also be explained by selection. It is possible that favorable alleles for different traits were selected, resulting in a high degree of LD on longer intermarker distances, even extending to inter chromosome pairs of SNP. Another potential reason for high r^2^ values when intermarker distance was large is assembling errors, potentially explaining the phenomenon on chromosome 1.

The average (SD) r^2^ between all pairwise SNPs contained on the same chromosome with intermarker distance greater than or equal to 0.10 and lower than 0.20 Mb was 0.1033 (0.0807) across all chromosomes. Zhao *et al*. observed r^2^ values equal to 0.044, 0.132 and 0.158 in Sunite, German Mutton Merino and Dorper sheep, respectively, in the same marker distance interval^36^. Additionally, García-Gámez *et al*. observed r^2^ equals to 0.086 for SNP also within the same marker distance interval in a Spanish Churra sheep population^28^. Similarly, Chitneedi *et al*. observed the average of 0.066 for r^2^ in Spanish Churra sheep using the high-density imputed genotypes^29^.

Using LD categories defined by Espigolan *et al*., Table 2 shows the average intermarker distances between pairwise SNPs exhibiting low LD (r^2^ ≤ 0.16), medium LD (0.16 < r^2^ < 0.70), and high LD (r^2^ > 0.70)^24^. Higher levels of r^2^ (greater than 0.70) were found at distances between markers smaller than 0.768 Mb with 3,296 combinations of SNPs (0.01% of all combinations). For medium levels of r^2^ (0.16 to 0.70), distances lower than 5.277 Mb were observed with 273,659 combinations of SNPs (0.849%). Considering low levels of r^2^ (lower than 0.16) distances found were higher than 15.110 Mb with 31,939,376 combinations of SNPs (99.140%).

**Table 2:** Mean intermarker distance and frequency for each category of linkage disequilibrium (high, medium and low) according to r^2^ metrics.

Chr	High			Medium			Low		
	Mean¹	Dist^2^	Freq³	Mean	Dist	Freq	Mean	Dist	Freq
OAR1	0.847	0.243	0.004	0.240	4.798	0.434	0.009	100.697	99.563
OAR2	0.850	0.463	0.009	0.248	4.518	0.669	0.011	63.832	99.323
OAR3	0.849	0.389	0.013	0.247	3.929	1.010	0.013	41.975	98.976
OAR4	0.847	0.158	0.010	0.244	4.370	0.984	0.014	41.952	99.006
OAR5	0.846	0.146	0.012	0.245	4.001	0.917	0.013	39.375	99.071
OAR6	0.848	0.520	0.007	0.242	3.899	0.724	0.013	42.614	99.270
OAR7	0.860	0.128	0.009	0.241	3.347	0.797	0.013	36.970	99.194
OAR8	0.844	0.171	0.011	0.240	4.007	0.913	0.014	33.116	99.076
OAR9	0.848	0.299	0.013	0.248	4.062	1.172	0.015	34.267	98.815
OAR10	0.842	0.768	0.039	0.259	5.277	1.929	0.018	27.292	98.033
OAR11	0.837	0.264	0.018	0.246	2.573	1.047	0.014	22.343	98.935
OAR12	0.849	0.237	0.011	0.244	3.355	1.129	0.015	28.272	98.860
OAR13	0.855	0.147	0.014	0.242	3.893	1.023	0.014	30.061	98.964
OAR14	0.849	0.119	0.016	0.252	2.588	1.039	0.014	22.174	98.945
OAR15	0.843	0.280	0.017	0.247	3.400	1.094	0.014	29.842	98.889
OAR16	0.813	0.408	0.036	0.268	4.708	2.056	0.016	26.320	97.908
OAR17	0.862	0.142	0.014	0.243	3.605	1.241	0.015	25.775	98.745
OAR18	0.8510	0.204	0.015	0.248	3.041	1.174	0.015	24.634	98.811
OAR19	0.835	0.222	0.019	0.246	2.766	1.238	0.016	21.592	98.743
OAR20	0.826	0.432	0.012	0.244	3.518	1.696	0.018	18.814	98.292
OAR21	0.846	0.104	0.022	0.243	2.980	1.823	0.019	17.715	98.154
OAR22	0.850	0.191	0.027	0.251	3.052	1.575	0.017	18.690	98.398
OAR23	0.873	0.129	0.010	0.235	3.796	1.360	0.017	22.134	98.630
OAR24	0.863	0.054	0.016	0.242	2.281	1.352	0.017	15.110	98.632
OAR25	0.872	0.094	0.022	0.244	2.949	1.697	0.018	16.127	98.280
OAR26	0.834	0.168	0.019	0.252	2.530	1.855	0.017	16.903	98.126

Low LD (*LD* $\le$ 0.16), medium LD (0.16 < *LD* < 0.70) and high LD (*LD* ≥ 0.70) for r^2^. ¹Mean r^2^ estimated from each pairwise combination of SNPs on each chromosome of interval. ^2^Intermarker distance for respective category between two by two marker (low, medium or high) (Mb), and ^³^Frequency of SNP number in each category, percentage (%).

### Relationship between linkage disequilibrium, inbreeding coefficient and effective population size

The relationships between r^2^, |D′|, MAF, F, and *N*_*e*_ are reported in Table 1. The mean MAF was similar across all chromosomes. The correlation between the two measures of LD was 0.75 when LD was estimated between adjacent SNP and 0.97 when estimated among all pairwise SNP. Although |D′| tends to overestimate LD values compared to r^2^ as reported by Zhao *et al*.^37^, both LD metrics exhibited the same behavior (Table 1). This is expected since these metrics are defined similarly as a function of allele frequency. The differences between the two metrics (r^2^ and |D′|) are related to the weight applied to the allele frequencies. Given |D′| is entirely dependent on the frequency of the alleles, |D′| possibly inflates LD estimates^37^. On the other hand, the r^2^ proposed by Hill and Robertson^7^ aims to reduce this frequency dependence.

According to Hill and Robertson^7^, LD (numerator of r^2^) and F have a linear relationship as shown in the equation below^7^. In a population under selection, the number of homozygotes tends to increase for many favorable alleles. Consequently, the inbreeding coefficient and LD between these selected alleles increase^7^.

1 $$E({D}^{2})=\frac{1}{15}{p}_{0}(1-{p}_{0}){q}_{0}(1-{q}_{0})[6(1-F)-5{(1-F)}^{3}-\,{(1-F)}^{6}]$$

where ${D}^{2}={({\rho }_{AB}-{\rho }_{A}{\rho }_{B})}^{2}$ and is the numerator of r^2^, ${\rho }_{A}\,\,$is the probability of allele A at marker 1, ${\rho }_{B}$ is the probability of allele B at marker 2, and ${\rho }_{AB}$ is a probability of the pair of AB markers; ${p}_{0}$ and ${q}_{0}$ are the frequency of A and B alleles, respectively, in generation zero or with initial equilibrium. A positive relationship (0.22) was observed between the D^2^ estimated by equation (1) as a function of inbreeding coefficients and the average D^2^ observed between adjacent SNPs on each the chromosome. A possible justification for the low correlation could be the relatively limited number of SNPs per chromosome on the panel used in the current study. The SNPs contained on the panel used herein covers only 299.6 Mb out of a total of 2,615.52 Mb, equivalent to 11% of the sheep genome. However, a few negative values were observed (e.g., −0.08) when estimating the correlation between D^2^ estimated by F (equation (1)) and average D^2^ between all pairwise SNPs on the chromosome. Additionally, equation (1) was derived under the assumption of finite and natural populations^7^.

The expectation of D at generation *t* can be derived from *c* (the recombination rate) and ${N}_{e}$. This is given by^38^:

2 $$E({D}_{t})=(1-c)(1-\frac{1}{2{N}_{e}})E({D}_{t-1})$$

A negative correlation between D, which is the numerator of |D′|, and both r^2^ and effective size (*N*_*e*_) is expected. Considering *N*_*e*_ as an indicator of selection, lower *N*_*e*_ values are a result of high selection pressure, and consequently a reduction in the number of breeding animals and genetic diversity. A negative relationship between average LD between all pairwise SNPs on a chromosome and *N*_*e*_ was observed (−0.16), as expected. However, the correlation between average LD between adjacent SNPs and *N*_*e*_ was positive (0.35). One potential reason for the observed discrepancy is the fact that *N*_*e*_ was estimated based on the LD between all pairwise SNPs rather than LD between adjacent SNPs. For instance, Lindblad-Toh *et al*. also observed that the effective population size and the inbreeding coefficient were reduced during dog domestication, resulting in a decrease of LD^39^.

### Haplotype blocks

The construction of haplotypes with only two (frequency = 1,879) to twenty-one (frequency = 1) markers was consistent with the low LD among pairwise SNP reported in this study. The mean size of haplotype blocks and the frequency of the number of SNPs for each chromosome are reported in Table 3. Short haplotype blocks in common among breeds have been observed by others^17^. The average distance (SD) between markers that formed the haplotype blocks was 0.04 (0.033) Mb. Considering the size of the sheep genome and the average distance between SNP that formed the haplotype blocks, it was possible to indirectly infer the minimum number of markers needed for genomic analyses, which was 61,415 SNPs. However, due to the high standard deviation of the distance between markers that formed the haplotype, it is important to use this number with caution.

**Table 3:** Summary of mean and standard deviation (SD) of intermarker distance in haplotype blocks for each chromosome and frequency of haplotype blocks size.

Chr	Mean blocks size (SD) (Mb)	Number of markers on haplotype block										**∑**
		2	3	4	5	6	7	8	9	10	21	
OAR1	2.278 (0.8138)	235	9	17	6	2		1				270
OAR2	2.516 (1.2153)	220	9	22	18	1	3			2		275
OAR5	2.447 (1.0964)	178	8	15	10	3	1	2				217
OAR6	2.432 (0.8914)	93	5	14	6							118
OAR5	2.367 (0.9296)	91	5	7	3	3						109
OAR6	2.215 (0.6147)	93	7	5	2							107
OAR7	2.241 (0.8413)	97	3	4	3			1				108
OAR8	2.363 (0.9605)	77	4	4	3	3						91
OAR9	2.225 (0.87058)	100	4	5		1			1			111
OAR10	2.798 (2.3260)	72	5	5	8	1	1			1	1	94
OAR11	2.292 (0.7978)	41	2	4		1						48
OAR12	2.325 (0.7425)	66	3	10	1							80
OAR13	2.557 (1.0882)	47	1	7	5	1						61
OAR14	2.317 (0.7225)	33	4	3	1							41
OAR15	2.540 (0.9972)	47	3	8	5							63
OAR16	2.387 (0.9470)	52	1	5	3	1						62
OAR17	2.270 (0.7450)	54	4	2	3							63
OAR18	2.367 (0.9724)	42	1	2	3	1						49
OAR19	2.314 (0.9485)	45		4	1		1					51
OAR20	2.325 (0.7642)	33	2	4	1							40
OAR21	2.344 (0.8273)	26	3	1	2							32
OAR22	2.232 (0.6873)	49	3	2	2							56
OAR23	2.531 (0.9153)	23	2	6	1							32
OAR24	2.960 (1.6452)	16	1	5	2				1			25
OAR25	2.286 (1.0167)	32		1	1		1					35
OAR26	2.167 (0.7071)	17			1							18

Chr: chromosome; SD: standard deviation; **∑**: sum of number of markers on haplotype block.

## Conclusions

The extent of LD among adjacent markers for the Santa Inês breed resembled those of previously reported results in other breeds of domesticated sheep. The mean LD values between all SNP pairs on each chromosome were consistent with domestic and wild sheep (*Ovis canadensis* and *Ovis dalli*) and they were lower than the estimates reported in other species. The findings reported in this study will be useful to provide a theoretical reference in determining the number of markers needed for future GS and GWAS in Santa Inês sheep.

## Methods

### Animal resources, genotyping and quality control

All experimental procedures employed in the present study that relate to animal experimentation were performed in accordance with the resolution number 07/2016 approved by Institutional Animal Care and Use Committee Guidelines from the School of Veterinary Medicine of University Federal of Bahia – UFBA and sanctioned by the president Prof. Claudio de Oliveira Romão to ensure compliance with international guidelines for animal welfare.

The dataset included the genotypes of 396 animals from the Santa Inês sheep breed collected between 2016 and 2017. These animals were fed in confinement for 54 to 92 days on average, during four different periods with slightly different nutritional management. This herd is located at the Experimental Farm of São Gonçalo dos Campos, the city of São Gonçalo dos Campos, Bahia, Brazil, and it is associated with the Federal University of Bahia (UFBA).

**Figure 3:**
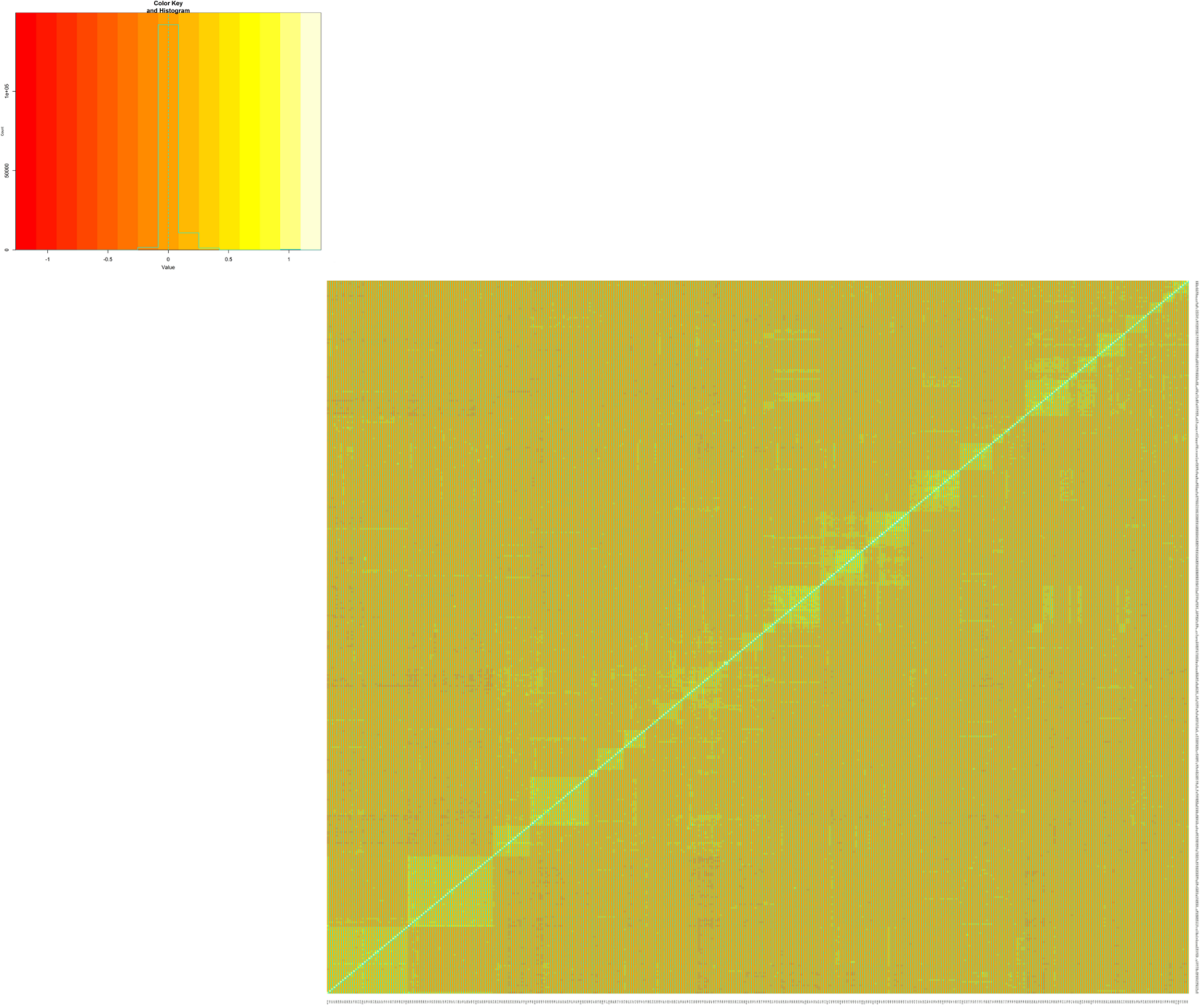
Hierarchically clustered heatmap of the genomic relationship among the individuals. At the top left, there is a histogram (green line) of the number of pairs of individuals (y axis = count) at each relationship degree (x axis = value). A vertical dashed green line is on the relationship degree equal to zero. At the bottom right, there is a heatmap of the relationship among the individuals. In both the histogram and the heatmap, the color gradient from dark red to light yellow represents the variation of the relationship degree from low to high, respectively.

To characterize the Santa Inês sheep population, the relationship between animals was estimated using a genomic relationship matrix, G, as described in VanRaden (2008)^40^. The G matrix was constructed by using the PREGSF90 software in the BLUPF90 package^41–43^. The average relationship between animals (SD) was 0.001 (0.0634), with minimum and maximum values equal to −0.135 and 0.934, respectively. The hierarchically clustered heatmap of the G matrix was constructed using the gplots R package^44^ and is presented in Fig. 3. The heatmap represents the relationship among individuals, with darker shades (red) representing low relationship between animals and lighter tones (light yellow) representing a high degree of relationship. The blocks observed in the heatmap represent individuals with stronger degrees of relationship than the overall mean relationship. By analyzing each block, we observed an overall relationship mean (standard deviation) within all blocks equal to 0.004 (0.0606), varying from −0.023 (0.0291) to 0.079 (0.1514). Random blocks with darker tones within the Fig. 3, for example, showed a lower mean (standard deviation) degree of relationship, with value equal to 0.001 (0.0555). None of the blocks can be considered as an exclusively full-sib or half-sib group^45^, although they include full-sib and half-sib relationships. Inside the most defined diagonal block, for example, 13 full-sib animal pairs and 350 half-sib animal pairs are represented. In the population as a whole, there are one twin animal pair, 38 full-sib animal pairs and 3,089 half-sib animal pairs. The structure of this population can be observed by a distribution printed into the left of Fig. 3, which presents the frequency of pairs by relationship degree. The major density of animal pairs is near zero, representing the overall low relationship among them. It is also possible to observe higher density of animal pairs above zero, closely to 0.25, 0.5 and 1.0, representing the half-sibs, full-sibs and twins as well as a mass lower than zero. The genetic structure of sampling might influence the LD results. For instance, a population with an elevated level of relationship probably will also have a higher level of inbreeding and, consequently, a higher LD level. Therefore, the complex breeding history of Santa Inês may have influenced the estimates of LD.

DNA was extracted from tissue samples of the *Longissimus dorsi* muscle collected from the left hemi-carcass and stored in 2.0 milliliter (ml) Eppendorf tubes. DNA extraction was performed according to protocols for lysis buffer and RNase. A high-density SNP panel (Illumina High-Density Ovine SNP BeadChip®) containing 54,241 SNP was used for genotyping. Chromosomal coordinates for each SNP were obtained from the ovine genome sequence assembly, Oar_v3.1.

Quality control (QC) of the genomic data was performed by the GenABEL R package^46^ for LD analyses^47^. The PREGSF90 interface of the BLUPF90 program^41–43^ was used to edit the genomic data for F, *N*_*e*_, MAF, and haplotype analyses. SNPs with a call rate lower than 0.90, MAF lower than 0.05 and p-value lower than 0.1 for the Hardy-Weinberg Equilibrium Chi-square test were excluded. One sample with a call rate lower than 0.9 was also removed. Table 4 summarizes the number of SNPs per chromosome before and after QC. We considered only the autosomal chromosomes (OAR1 to OAR26) in this study resulting in 38,168 SNPs retained for further analysis.

**Table 4:** The number of SNPs per chromosome before and after quality control.

Chr	N° ${{\rm{SNPs}}}_{i}$	N° ${{\rm{SNPs}}}_{f}$
1	5931	4392
2	5475	4020
3	5009	3606
4	2681	1976
5	2364	1723
6	2593	1979
7	2253	1664
8	2058	1521
9	2142	1539
10	1739	1319
11	1181	860
12	1724	1245
13	1697	1214
14	1175	836
15	1695	1223
16	1581	1090
17	1421	1070
18	1414	1011
19	1249	887
20	1149	818
21	899	654
22	1098	758
23	1129	835
24	742	524
25	1002	731
26	925	673

Chr: chromosome; N° SNPs_i_: SNP count before quality control; N° SNPs_*f*_: SNP count after quality control.

### Inbreeding coefficient and effective population size

Inbreeding coefficient (F) was calculated as a function of the expected and observed homozygote difference by using the PLINK software^48^. This is given by

3 $${F}_{i}=\frac{({O}_{i}-{E}_{i})}{({L}_{i}-{E}_{i})}$$

where ${F}_{i}$ is the estimated inbreeding coefficient of the *i*^*ih*^ animal; ${O}_{i}$ is the number of homozygous loci observed in the *i*^*ih*^ animal, ${E}_{i}$ is the number of homozygous loci expected and ${L}_{i}$ is the number of genotyped autosomal loci^48^.

Effective population size (*N*_*e*_) was obtained by the SNeP software^49^. This software provides a history of the effective population size, that is, the number of past generations based on the relationship between *N*_*e*_, linkage disequilibrium represented by r^2^, and recombination rate (*c*) by using the following equation^50^.

4 $$E[{r}^{2}]={(1+4{N}_{e}c)}^{-1}$$

Therefore, by solving equation (4), we have:

5 $${N}_{e(t)}={(4f({c}_{t}))}^{-1}(E{[{r}^{2}|{c}_{t}]}^{-1}-\alpha )$$

where ${N}_{e(t)}$ is the effective population size at generation *t*, which is ${(4f({c}_{t}))}^{-1}$^51^; ${c}_{t}$ is the recombination rate in generation *t* which is proportional to the physical distance between markers, r^2^ is LD, and $\alpha$ ${\rm{is}}$ the adjustment for mutation rate. The parameter α can assume three different values: $1,\,2$ or $2.2$^52^. When we consider $\alpha$ equal to 1, ${N}_{e}c$ tends towards 0 and we assume that there is no selection or mutation. On the other hand, when mutation does occur, the parameter $\alpha$ can be equal to 2 or 2.2. The value of 2.2 comes from the result of the equilibrium expression $\frac{E[{({\rho }_{AB}-{\rho }_{A}{\rho }_{B})}^{2}]}{E[{\rho }_{A}(1-{\rho }_{A}){\rho }_{B}(1-{\rho }_{B})]}$ that was equal to $\frac{5}{11}$. In this expression, ${\rho }_{A}\,\,$is the probability of allele A at marker (or SNP) 1, ${\rho }_{B}$ is the probability of allele B at marker (or SNP) 2, and ${\rho }_{AB}$ is a probability of the pair of AB markers; following Ohta & Kimura^52^. Tenesa *et al*. proposed $\alpha$ equal to two^53^.

In our study, the ${N}_{e}\,\,$by chromosome was the result of a harmonic mean due to a relatively small number of SNPs in each chromosome. The physical distance was transformed to genetic distance considering one Mb as one centimorgan (cM).

### Linkage disequilibrium analysis

The estimation of LD was performed in two ways for each chromosome: (1) between neighboring pairs of SNPs (adjacent SNPs) and (2) pairwise combination of all SNPs (pairwise SNPs) using the function LD in the R package genetics^47,54^. The |D′| is a scale of the frequency difference of the allele pairs AB, where A is the allele of the marker (or SNP) 1, and B the allele of the marker 2, and the expected frequency of each allele separately. |D′| parameter ranges from 0 to 1 and it is given by^55^:

6 $${D}^{\text{'}}=\frac{D}{Dmax}$$

And

7 $$D=\,{\rho }_{AB}-{\rho }_{A}{\rho }_{B}$$

Where

8 $$\,\{\begin{array}{c}D > 0,\,{D}_{max}=\,{\rm{\min }}({\rho }_{A}{\rho }_{b},{\rho }_{a}{\rho }_{B})\\ D < 0,\,{D}_{max}=\,{\rm{\max }}(-\,{\rho }_{A}{\rho }_{B},-{\rho }_{a}{\rho }_{b})\end{array}\,\}$$

Here ${\rho }_{A}\,\,$is the probability of allele A at marker 1, ${\rho }_{a}$ is the probability of allele a at marker 1, ${\rho }_{B}$ is the probability of allele B at marker 2, ${\rho }_{b}$ is the probability of allele b at marker 2, and ${\rho }_{AB}$ is a probability of the pair of AB markers. Maximum likelihood was used to estimate ${\rho }_{AB}$ because genotype AB/ab is not distinguishable from genotype aB/Ab^56^.

The squared correlation between the markers, given by r^2^, is expressed as^7^:

9 $${r}^{2}=\frac{{D}^{2}}{({\rho }_{A}{\rho }_{a}{\rho }_{B}{\rho }_{b})}$$

where $\,{D}^{2}={({\rho }_{AB}-{\rho }_{A}{\rho }_{B})}^{2}$, ${\rho }_{A}\,\,$is the probability of allele A at marker 1, ${\rho }_{a}$ is the probability of allele a at marker 1, ${\rho }_{B}$ is the probability of allele B at marker 2, and ${\rho }_{b}$ is the probability of allele b at marker 2.

In total, four LD estimates were obtained: (1) |D′| between adjacent SNPs; (2) |D′| between all pairwise SNPs; (3) r^2^ between adjacent SNPs; and (4) r^2^ between all pairwise SNPs.

### Haplotype blocks

The haplotype blocks were identified by following the approach suggested by Gabriel *et al*.^57^ which was implemented via PLINK^48^. Blocks were partitioned according to whether the upper and lower confidence limits on estimates of pairwise |D′| measure fall within certain threshold values. The desired SNP panel density was estimated by the ratio of the megabase pair over the entire ovine genome and distance between markers that composed the haplotype blocks.

### Data availability

Data are available on request.

### Declarations

All experimental procedures involving sheep were approved by the Institutional Animal Care and Use Committee Guidelines from School of Veterinary Medicine of University Federal of Bahia – UFBA and sanctioned by the president Prof. Claudio de Oliveira Romão (n° 07/2016). All experiments were performed in accordance with relevant guidelines and regulations.

## Electronic supplementary material

Supplementary figures and supplementary tables
