# Supplementary material for: Linkage disequilibrium in Brazilian Santa Inês breed, Ovis aries
Source: Sci Rep. 2018 Jun 11;8:8851. doi: 10.1038/s41598-018-27259-7 (PMC5995818; doi:10.1038/s41598-018-27259-7)
Supplement: Supplementary file 1 — Supplementary figures and supplementary tables [file 41598_2018_27259_MOESM1_ESM.pdf]

Supplementary Figures

Linkage disequilibrium in Brazilian Santa Inês breed, *Ovis aries*

Amanda Botelho Alvarenga[1], Gregori Alberto Rovadoscki[1], Juliana Petrini[1], Luiz Lehmann Coutinho[1], Gota Morota[2], Matthew L. Spangler[2], Luís Fernando Batista Pinto[3], Gleidson Giordano Pinto Carvalho[3], & Gerson Barreto Mourão[1*]

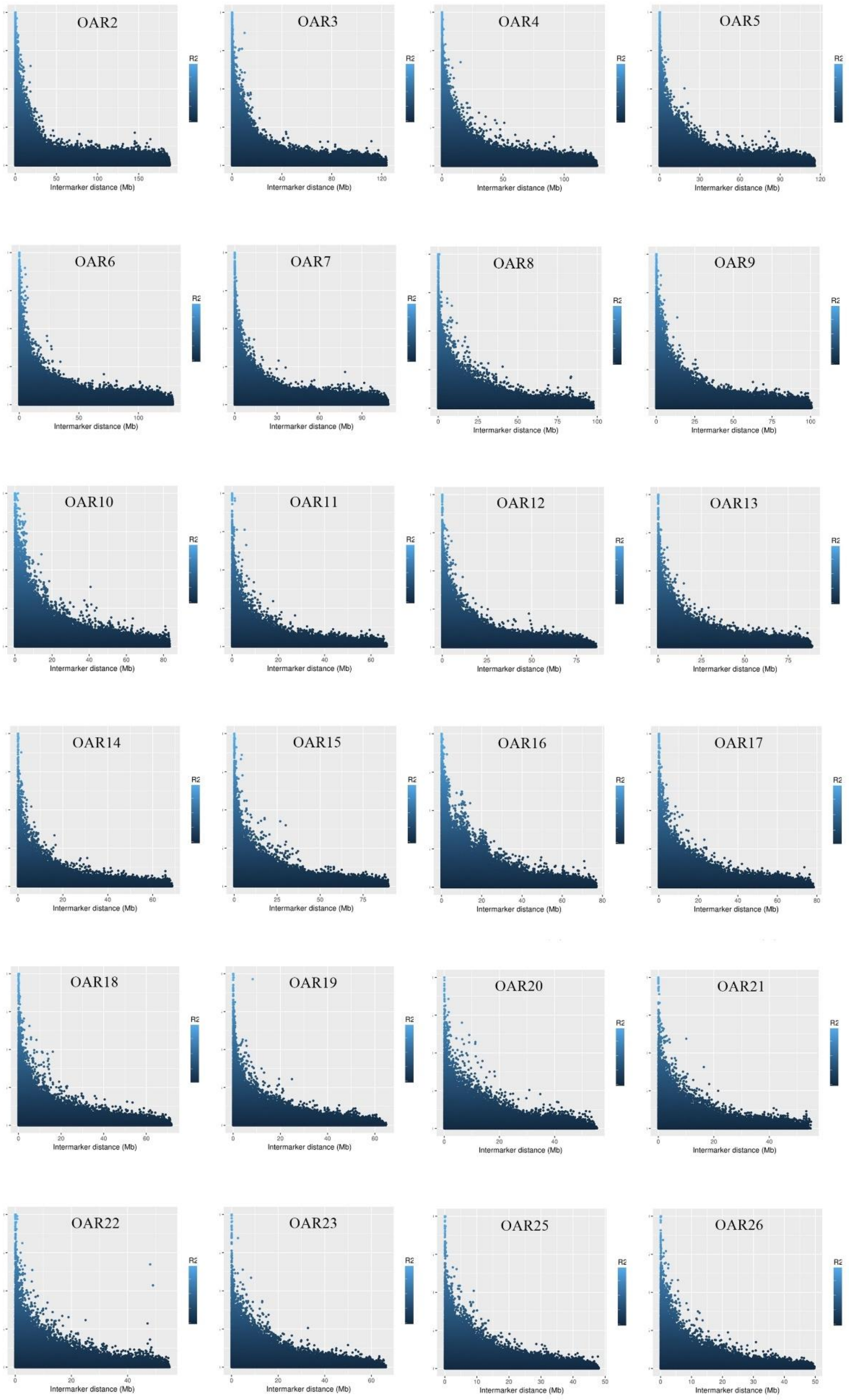

Supplementary Fig. S1. LD measured by r^2 plotted as a function of intermarker distance (Mb) for chromosomes.

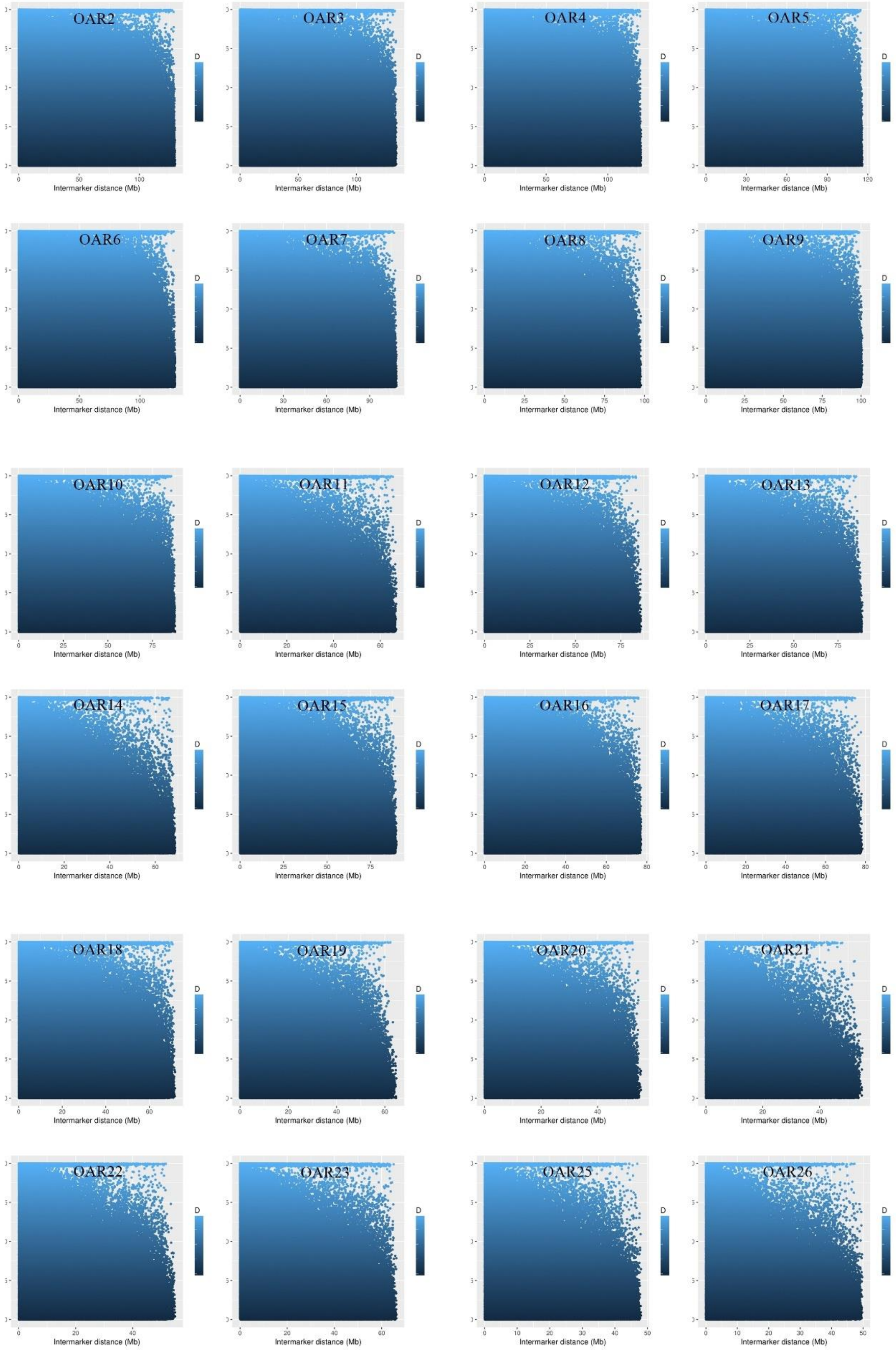

Supplementary Fig. S2. LD measured by |D'| plotted as a function of intermarker distance (Mb) for chromosomes.

Supplementary Tables

Linkage disequilibrium in Brazilian Santa Inês breed, *Ovis aries*

Amanda Botelho Alvarenga[1], Gregori Alberto Rovadoscki[1], Juliana Petrini[1], Luiz Lehmann Coutinho[1], Gota Morota[2], Matthew L. Spangler[2], Luís Fernando Batista Pinto[3], Gleidson Giordano Pinto Carvalho[3], & Gerson Barreto Mourão[1*]

Supplementary Table. S1. Summary of average linkage disequilibrium (r²) between all pairwise SNP pairs by chromosome from the current study, Al-Mamum et al. (2015)[13], Miller et al. (2011)[4] and García-Gámez et al. (2012)[28] .

Chr	Alvarenga et al.	Al-Mamum et al. 2015					Miller et al. 2011	García-Gámez et al. 2012
	r^2 pairwise	r^2 pairwise					r^2 pairwise	r^2 pairwise
		BL	PD	MER	BxM	BxMxP		
1	0.010	0.008	0.008	0.005	0.006	0.008	0.035	0.006
2	0.011	0.009	0.009	0.006	0.007	0.008	0.037	0.008
3	0.011	0.009	0.009	0.006	0.007	0.009	0.035	0.008
4	0.016	0.010	0.013	0.006	0.007	0.010	0.039	0.010
5	0.015	0.010	0.012	0.006	0.007	0.010	0.048	0.012
6	0.014	0.012	0.012	0.006	0.008	0.010	0.036	0.013
7	0.015	0.011	0.012	0.006	0.008	0.011	0.045	0.009
8	0.016	0.011	0.014	0.007	0.008	0.010	0.051	0.011
9	0.018	0.011	0.012	0.006	0.008	0.010	0.035	0.009
10	0.020	0.014	0.015	0.007	0.009	0.012	0.042	0.012
11	0.017	0.011	0.014	0.007	0.008	0.010	0.044	0.012
12	0.017	0.012	0.013	0.007	0.008	0.010	0.043	0.011
13	0.017	0.012	0.013	0.007	0.008	0.011	0.046	0.009
14	0.017	0.011	0.014	0.007	0.008	0.010	0.035	0.010
15	0.017	0.011	0.014	0.007	0.008	0.011	0.025	0.010
16	0.022	0.011	0.015	0.007	0.008	0.012	0.071	0.011
17	0.018	0.013	0.015	0.007	0.009	0.011	0.052	0.011
18	0.018	0.012	0.015	0.007	0.008	0.011	0.060	0.010
19	0.019	0.012	0.014	0.008	0.008	0.011	0.100	0.009
20	0.022	0.012	0.016	0.007	0.008	0.011	0.085	0.015
21	0.023	0.013	0.017	0.007	0.009	0.011	0.034	0.010
22	0.021	0.014	0.019	0.007	0.009	0.012	0.006	0.010
23	0.020	0.012	0.016	0.007	0.008	0.012	0.041	0.013
24	0.020	0.013	0.014	0.008	0.009	0.011	0.026	0.013

25	0.018	0.014	0.017	0.008	0.009	0.012	0.030	0.014
26	0.022	0.013	0.018	0.007	0.009	0.012	0.041	0.014
	**0.018**	**0.012**	**0.014**	**0.007**	**0.008**	**0.011**	**0.042**	**0.011**

Chr: chromossome; BL: Border Leicester; PD: Poll Dorset; MER: Merino; MxB: crosses of Merino and Border Leicester; MxBxP: MxB crossed to Poll Dorset.

Supplementary Table. S2. Summary of average linkage disequilibrium (r²) between adjacent SNP pairs by chromosome from the current study, Al-Mamum et al. (2015)[13], Mastrangelo et al. (2014)[31] and Brito et al. (2017)[12].

Chr	Alvarenga et al.	Al-Mamum et al. 2015					Mastrangelo et al. 2014			Brito et al. 2017	Mastrangelo et al. 2017
	r^2 adjacent	r^2 adjacent					r^2 adjacent			r^2 adjacent	r^2 adjacent
		BL	PD	MER	BxM	BxMxP	VDB	COM	PIN		
1	0.172	0.210	0.196	0.130	0.142	0.147	0.162	0.161	0.132	0.263	0.240
2	0.192	0.226	0.208	0.143	0.156	0.153	0.182	0.189	0.148	0.275	0.240
3	0.183	0.222	0.204	0.134	0.150	0.148	0.170	0.172	0.140	0.276	0.229
4	0.181	0.211	0.202	0.137	0.146	0.151	0.171	0.164	0.139	0.267	0.222
5	0.169	0.212	0.192	0.124	0.134	0.143	0.150	0.154	0.127	0.263	0.214
6	0.155	0.210	0.190	0.124	0.142	0.141	0.158	0.165	0.131	0.262	0.242
7	0.167	0.229	0.205	0.129	0.153	0.155	0.151	0.158	0.137	0.264	0.214
8	0.165	0.216	0.196	0.128	0.142	0.144	0.164	0.155	0.133	0.260	0.214
9	0.166	0.211	0.187	0.128	0.137	0.141	0.167	0.170	0.137	0.259	0.212
10	0.191	0.246	0.210	0.148	0.166	0.162	0.168	0.179	0.148	0.267	0.259
11	0.152	0.175	0.173	0.111	0.114	0.123	0.155	0.155	0.118	0.271	0.214
12	0.157	0.219	0.182	0.126	0.138	0.145	0.165	0.157	0.131	0.257	0.212
13	0.169	0.209	0.198	0.137	0.139	0.142	0.151	0.170	0.133	0.282	0.221
14	0.157	0.184	0.182	0.110	0.117	0.116	0.133	0.134	0.114	0.261	0.217
15	0.169	0.195	0.200	0.124	0.130	0.138	0.149	0.153	0.129	0.264	0.234
16	0.194	0.185	0.192	0.121	0.124	0.138	0.149	0.157	0.124	0.249	0.190
17	0.155	0.218	0.188	0.117	0.142	0.135	0.155	0.152	0.125	0.247	0.213
18	0.160	0.195	0.188	0.127	0.131	0.140	0.149	0.156	0.128	0.263	0.217
19	0.172	0.204	0.192	0.137	0.141	0.141	0.156	0.167	0.139	0.260	0.226
20	0.148	0.180	0.166	0.109	0.115	0.117	0.150	0.138	0.117	0.255	0.206
21	0.157	0.188	0.182	0.108	0.125	0.122	0.152	0.151	0.124	0.246	0.191
22	0.173	0.206	0.196	0.118	0.133	0.142	0.154	0.155	0.125	0.254	0.192
23	0.142	0.174	0.184	0.108	0.107	0.118	0.156	0.140	0.107	0.245	0.195
24	0.135	0.195	0.162	0.109	0.118	0.122	0.134	0.134	0.108	0.262	0.189
25	0.166	0.197	0.195	0.114	0.124	0.136	0.141	0.142	0.116	0.249	0.208

26	0.165	0.184	0.176	0.107	0.115	0.120	0.143	0.137	0.116	0.244	0.192
	**0.166**	**0.204**	**0.190**	**0.123**	**0.134**	**0.138**	**0.155**	**0.156**	**0.128**	**0.263**	**0.215**

Chr: chromossome; BL: Border Leicester; PD: Poll Dorset; MER: Merino; MxB: crosses of Merino and Border Leicester; MxBxP: MxB crossed to Poll Dorset; VDB: Valle del Belice; COM: Comisana; PIN: Pinzirita.
